# Metabolic syndrome biomarkers and early breast cancer in Saudi women: evidence for the presence of a systemic stress response and/or a pre-existing metabolic syndrome-related neoplasia risk?

**DOI:** 10.1186/1471-2407-13-54

**Published:** 2013-02-04

**Authors:** Majed S Alokail, Nasser Al-Daghri, Amal Abdulkareem, Hossam M Draz, Sobhy M Yakout, Abdullah M Alnaami, Shaun Sabico, Amal M Alenad, George P Chrousos

**Affiliations:** 1 Biomarkers Research Program, Biochemistry Department, College of Science, King Saud University, Riyadh, 11451, Kingdom of Saudi Arabia (KSA; 2 Center of Excellence in Biotechnology, King Saud University, Riyadh, 11451, KSA; 3 Department of Surgery, College of Medicine, King Saud University, Riyadh, 11472, KSA; 4 Department of Biochemistry, National Research Centre, Cairo, 12311, Egypt; 5 School of Biological Sciences, Life Science Building 85, University of Southampton, Southampton, SO17 1BJ, UK; 6 First Department of Pediatrics, Athens University Medical School, Athens, 11527, Greece; 7 Department of Biochemistry, College of Science, King Saud University, PO Box 2455, Riyadh, 11451, Kingdom of Saudi Arabia

**Keywords:** Breast cancer, Saudi women, Adiponectin, Leptin, HDL

## Abstract

**Background:**

Obesity has been linked to many adverse health consequences, including breast cancer. This study aims to determine adipocytokine and other biological changes in recently diagnosed breast cancer patients before therapy is started.

**Methods:**

A total of 109 female Saudi subjects [56 newly diagnosed, treatment-naïve, histologically-confirmed breast cancer cases and 53 age- and BMI-matched controls] were enrolled in this study. Anthropometric data were collected. Serum insulin, adipocytokines and plasminogen activator inhibitor-1 (PAI-1) concentrations were measured using a customized multiplex Luminex assay. Hypersensitive C-Reactive Protein (CRP), tumor necrosis factor-alpha (TNF-α), and angiotensin II (ANG II) were measured using ELISA.

**Results:**

A few days in the diagnosis, breast cancer subjects had significantly higher systolic blood pressure (*p* = 0.03), glucose (*p* = 0.01), triglycerides (*p* = 0.001), leptin (*p* = 0.044), resistin (*p* = 0.04), ANG II (*p* = 0.02), TNF-α (*p* = 0.045), and CRP (*p* = 0.04) than the controls. On the other hand, HDL (*p* = 0.01) and adiponectin (*p* = 0.02) were significantly lower in cancer subjects than controls. A significant association was found between elevated triglycerides (TG) and breast cancer [OR (95% CI), 6.1(1.8, 15.6), *p* = 0.004], as well as elevated ANG II [OR (95% CI), 5.2(1.2, 14.3), *p* = 0.03]. On the other hand, aPAI and HDL correlated negatively with breast cancer [OR (95% CI), 0.076(0.01, 0.34), *p* = 0.001; 0.30(0.09, 0.95), *p* 0.04, respectively].

**Conclusion:**

Circulating ANGII and triglycerides were positively associated with early breast cancer. In contrast, HDL-cholesterol correlated negatively with ANG II and aPAI in these patients. This suggests that patients with recently diagnosed breast cancer have biochemical changes consistent with an activated stress response and/or that patients with metabolic syndrome manifestations have a higher risk of developing this disease.

## Background

Overweight and obesity, a global phenomenon, affects more than 1 billion adults, with 300 million being clinically obese [1]. Obesity has a major impact on the risk and prognosis of some of the more common forms of cancer, but also provides us with one of the few preventive interventions capable of making a significant impact on the cancer problem [2]. Weight increase and obesity in menopausal females have been identified as the most important prognostic risk factors for breast cancer in postmenopausal women [3]. Several studies have reported that at diagnosis of breast cancer, obese women exhibit an increase in lymph-node involvement and a higher propensity to develop distant metastases [4,5].

Breast cancer is the most commonly occurring female cancer in the industrialized world. Although early diagnosis has contributed to therapeutic success, breast cancer remains a major female health issue and its incidence is increasing in developing countries [3]. Genetic predisposition and environmental factors, such as a high fat diet and alcohol consumption, accompanied with a sedentary life style had been reported to cause an increase in breast cancer risk [3,6].

Metabolic Syndrome, including obesity and T2DM, are positively associated with an increased breast cancer risk [7,8]. These conditions are associated with changes in several hormonal systems, including insulin, estrogen, cytokines and growth factors [3]. Recent studies have linked breast cancer with insulin resistance [9-11]; metabolic syndrome (MetS) [12,13], and altered adipokine levels [14].

Alteration in adipocytokine production in obese subjects has been reported in several studies. Studies have shown that increased leptin and decreased adiponectin levels promote carcinogenesis of the breast [15-18]. It was also shown that adiponectin has prognostic significance in breast cancer recurrence [10]. In addition, obesity is being increasingly recognized as a form of systemic subclinical inflammation and, accordingly, an increased adipose tissue infiltration by immune cells producing inflammatory substances, including C-reactive protein (CRP) and tumor necrosis factor-alpha (TNF-α), which have a positive impact on the breast cancer development [19-21]. CRP is positively and negatively correlated with leptin and adiponectin levels, respectively [22,23]. Independent effect of CRP and alterations in the levels of both leptin and adiponectin were altogether accompanied by an increase in breast cancer risk incidence [24].

This aim of this study was to further examine adipocytokines and other metabolic and immune biomarkers of metabolic syndrome linked with obesity in patients with early breast cancer prior to therapy initiation.

## Methods

A case–control study was conducted by the Biomarkers Research Program (BRP), College of Science, King Saud University, Riyadh, Kingdom of Saudi Arabia (KSA). Ethical approval for the study was granted by the Ethics Committee of King Khalid Hospital, King Saud University, Riyadh, Kingdom of Saudi Arabia (KSA). A total of 109 female Saudi subjects, consisting of 56 newly diagnosed, histologically-confirmed breast cancer with no prior breast cancer treatment and 53 age- and BMI-matched controls were enrolled in this study. All of the control subjects were confirmed free from benign or malignant breast diseases and women with a personal or family history of any tumor was excluded. All subjects were free from acute medical conditions, including infections, at the time of inclusion. Control samples were matched according to the age and BMI of cases and were taken from an existing database from the Biomarkers Research Program. Written informed consent for the utilization of serum samples and personal information through a questionnaire for the purpose of research was obtained from all subjects.

### Anthropometrics

Anthropometric data were collected by a designated research nurse and physician: Height (to the nearest 0.5 cm), weight (to the nearest 0.1 kg), waist and hip circumference (measured using a standardized measuring tape in cm) in addition to systolic and diastolic blood pressure measurements. Body mass index (BMI) was calculated as kg/m^2^. WHR was also calculated as waist divided by hip circumference. Fasting blood samples from cases were extracted after diagnosis and prior to breast cancer therapy initiation. Blood was transferred immediately to a non-heparinized tube for centrifugation. Serum was then transferred to a pre-labeled plain tube, stored in ice, and delivered to the Biomarker Research Center in King Saud University on the same day.

### Metabolic measurements

Fasting serum samples were stored in a −20°C freezer prior to analysis. Serum glucose, triglycerides, total and HDL-cholesterol levels were measured by chemistry auto-analyzer (Konelab, Espoo, Finland) and concentrations of LDL-cholesterol were calculated using Friedwald's formula. Determination of serum insulin, leptin, adiponectin, resistin, and aPAI-1 was done using customized multiplex assay kits that utilize the Luminex® xMAP® Technology platform (Luminex Corporation, Texas, USA).

For parameters measured using the multiplex assay, the intra-assay variation was 1.4-7.9% and inter-assay variation of < 21%. Minimum detectable concentrations (MDC) were as follows: insulin 50.9 pg/ml; leptin 85.4 pg/ml; adiponectin 145.4 pg/ml; resistin 6.7 pg/ml; and PAI-1 1.3 pg/ml. The rest of the parameters were quantified using individual enzyme-linked immunosorbent assay kits (ELISA): CRP (Immunodiagnoztik AG, Germany) with an intra-assay variability of 5.5-6.0% and inter-assay variation of 11.6-13.8%; TNF-α (Biosource, Belgium); ANG-II (R and D Systems, MN).

### Statistical analysis

Data analysis was carried out using the Statistical Package for the Social Sciences (SPSS for Windows, version 16.0). Power calculation was done ascertaining differences in mean CRP levels between cases and controls. A total sample size of 88 has 80% power to detect a significance at α = 0.03. Data are expressed as mean ± standard deviation, while medians (inter-quartile range) were shown for non-normal continuous variables. Independent student t-test was used to compare group differences for normal parameters. For non-normal parameters, Mann–Whitney U-test was utilized. Multinomial logistic regression analysis was done using the presence of breast cancer as dependent variable and parameters of interest as independent variables adjusted for menopause and age of menarche. Partial correlation analysis was used to determine associations between variables of interest. Significance was set at p < 0.05. All statistical analyses were conducted using SPSS version 16.5 (Chicago, IL).

## Results

Table 1 highlights the general characteristics of our female subjects. Data revealed that breast cancer subjects had significantly higher systolic blood pressure (*p* = 0.03), glucose (*p* = 0.01), triglycerides (*p* = 0.001), leptin (*p* = 0.044), resistin (*p* = 0.04), Ang II (*p* = 0.02), TNF-α (*p* = 0.045), and CRP (*p* = 0.04) than controls. On the other hand, HDL (*p* = 0.01) and adiponectin (*p* = 0.02) were significantly lower in breast cancer subjects than controls. The rest of the comparisons were non-contributory.

**Table 1:** General characteristics of subjects

	**Control**	**Breast cancer**	**P value**
N	53	56	
Age (years)	43.1 ± 7.5	46.4 ± 11.3	0.10
Age at Menarche (years)	13.1 ± 1.0	12.9 ± 1.6	0.48
Age at 1^st^ Pregnancy (years)	21.0 ± 3.8	19.6 ± 4.3	0.19
Menopause (%)	9 (17)	22 (40)	0.01
Body Mass Index (kg/m^2^)	31.0 ± 5.4	31.4 ± 7.7	0.81
Systolic BP (mmHg)	112.3 ± 11.98	118.6 ± 15.5	0.03
Diastolic BP (mmHg)	73.3 ± 7.0	70.9 ± 10.5	0.22
SAD (cm)	22.1 ± 5.8	20.2 ± 8.3	0.30
Waist circumference (cm)	88.8 ± 18.8	96.3 ± 22.2	0.08
Hip circumference (cm)	106.5 ± 21.4	105.5 ± 18.2	0.81
Glucose (mmol/l)	5.4 ± 0.63	5.9 ± 1.2	0.01
Triglycerides (mmol/l)	1.3 ± 0.22	1.9 ± 0.38	0.001
Total Cholesterol (mmol/l)	4.7 ± 0.62	4.9 ± 1.0	0.27
LDL-Cholesterol (mmol/l)	3.6 ± 0.76	3.7 ± 1.0	0.51
HDL-Cholesterol (mmol/l)	0.86 ± 0.29	0.72 ± 0.26	0.01
C-Reactive Protein (ug/ml)	4.4 ± 0.11	7.5 ± 0.21	0.04
ANG II (ng/ml)	0.77 ± 0.15	0.99 ± 0.29	0.02
Leptin (ng/ml)	16.0 ± 2.2	25.6 ± 1.7	0.044
Adiponectin (ug/ml)	19.1 ± 1.2	14.8 ± 1.0	0.02
TNF-α (pg/ml)	4.6 ± 0.57	6.0 ± 0.75	0.045
aPAI (ng/ml)	14.6 ± 1.3	12.2 ± 2.8	0.08
Resistin (ng/ml)	15.2 ± 1.0	18.9 ± 1.2	0.04
Ca (mmol/l)	2.4 ± 0.23	2.3 ± 0.58	0.18
Pi (mmol/l)	1.2 ± 0.18	1.6 ± 0.34	<0.001

**Note**: Data presented as mean ± SD; P-value significant at < 0.05.

Table 2 shows odds ratios for breast cancer in relation to glucose, HDL, triglycerides, CRP, ANG II, adiponectin, leptin, TNF-α, aPAI, and resistin levels. A significant association was found between elevated levels of triglycerides and risk of breast cancer [OR (95% CI), 6.1 (1.8, 15.6), *p* = 0.004]. Significant associations were also found between elevated levels of ANG II and risk of developing breast cancer in females [OR (95% CI), 5.3 (1.2, 14.3), *p* = 0.03]. On the other hand aPAI and HDL had a protective effect with the risk of developing breast cancer [OR (95% CI), 0.076 (0.01, 0.34), *p* = 0.001; 0.30 (0.09, 0.95), *p* = 0.04, respectively].

**Table 2:** **Menopausal status and Age of menarche** -**adjusted Odds**-**ratio** [**confidence interval** (**CI**) **95**% **for Breast cancer in Relation to Glucose**, **HDL**, **Triglycerides**, **CRP**, **ANG II**, **Adiponectin** , **Leptin**, **TNF**-**a**, **aPAI**, **and Resistin levels**

	**Odds ratio(95% CI)**	**P-Value**
Glucose (mmol/l)	2.2 (0.68, 7.1)	0.63
Triglycerides (mmol/)	6.1 (1.8, 15.6)	0.004
HDL-Cholesterol (mmol/l)	0.30 (0.09, 0.95)	0.04
C-Reactive Protein (ug/ml)	2.1 (0.53, 8.1)	0.29
ANG II (ng/ml)	5.3 (1.2, 14.3)	0.03
Leptin (ng/ml)	1.6 (0.33, 7.9)	0.53
Adiponectin (ug/ml)	0.44 (0.12, 1.5)	0.19
Resistin (ng/ml)	1.9 (0.62, 5.7)	0.26
TNF-α (pg/ml)	1.59 (0.44, 5.7)	0.47
aPAI (ng/ml)	0.076 (0.01, 0.34)	0.001

**Note**: P-value significant at p < 0.05.

Linear regression analyses using CRP, ANG II, adiponectin, leptin, TNF-α, aPAI, and resistin as dependent variables in all subjects, controls and cases are shown in Table 3. Data showed that CRP was positively associated with BMI (r = 0.38, p = 0.001), waist (r = 0.44, p > 0.001), leptin (r = 0.44, p > 0.001) and negatively associated with adiponectin (r = −0.27, p = 0.02) and resistin (r = −0.26, p = 0.02). Adiponectin was negatively associated with waist (r = −0.35, p = 0.001), triglycerides (r = −0.26, p = 0.01), and CRP (r = −0.27, p = 0.02) and positively associated with HDL (r = 0.23, p = 0.03). Leptin was positively associated with BMI (r = 0.32, p = 0.006), waist (r = 0.23, p = 0.05), hips (r = 0.25, p = 0.04), CRP (r = 0.26, p = 0.04), and TNF-α (r = 0.23, p = 0.05). TNF-α was positively associated with triglycerides (r = 0.26, p = 0.01), leptin (r = 0.23, p = 0.05), aPAI-1 (r = 0.44, p < 001). aPAI-1 was positively associated with diastolic BP (r = 0.25, p = 0.03), glucose (r = 0.28, p = 0.01), and TNF-α (r = 0.44, p < 0.001) and negatively associated with cholesterol (r = −0.28, p = 0.02), LDL (r = −0.29, p = 0.008) and calcium (r = −0.61, p < 0.001). Resistin was positively associated with calcium (r = 0.36, p = 0.01) and negatively associated with CRP (r = −0.26, p = 0.02).

**Table 3:** **Correlation analysis using CRP**, **ANG II**, **Adiponectin**, **Leptin**, **TNF**-**α**, **aPAI and Resistin as dependent variables in all subjects**, **controls and cases**

	**CRP**	**ANG II**	**Leptin**	**Adiponectin**	**Resistin**	**TNF-α**	**aPAI**
	**All; Control; Cases**	**All; Control; Cases**	**All; Control; Cases**	**All; Control; Cases**	**All; Control; Cases**	**All; Control; Cases**	**All; Control; Cases**
Age (years)							
BMI (kg/m^2^)	0.38**; NS; 0.52**		0.32**; NS; 0.44**				
Systolic BP (mmHg)							NS; NS; 0.36*
Diastolic BP (mmHg)							0.25*; NS; NS
SAD (cm)							
Waist (cm)	0.44**; NS; 0.48**		0.23*; NS; NS	−0.35**; NS; -0.45**			
Hips (cm)	NS; NS; 0.35*		0.25*; NS; 0.44*				
Glucose (mmol/l)				NS; NS; -0.38*			0.28*; NS; NS
Total Cholesterol (mmol/l)							−0.28*; NS; NS
Triglycerides (mmol/l)				−0.26*; NS; NS		0.26*; NS; 0.28*	
HDL-Cholesterol (mmol/l)				0.23*; NS; NS			
LDL-Cholesterol (mmol/l)	NS; 0.38*; NS						−0.29**;-0.31*;
CRP (ug/ml)			0.26*; NS; 0.40*	−0.27*; -0.32*; NS	−0.26*; NS; -0.32*		
ANG II (ng/ml)							NS; 0.48**; NS
Leptin (ng/ml)	0.26*; NS; 0.41*					0.23*; NS; NS	
Adiponectin (ug/ml)	−0.27*; NS; NS			NS; 0.28*; NS		NS; 0.34*; NS	
Resistin (ng/ml)	−0.26*; NS; -0.32*						NS; 0.45**; NS
TNF-α (pg/ml)			0.23*; 0.34*;	NS; NS; -0.38*			0.44**; 0.45**; 0.58**
aPAI (ng/ml)		NS; 0.48*; NS		NS; 0.37**; NS		0.44**; 0.45**; 0.58**	
Ca (mmol/l)							−0.61**; NS; -0.75**
Pi (mmol/l)					0.36**; NS; NS		

**Note**: Only significant associations were presented; Values presented as coefficient R; Coefficients presented from left to right [All, Control, Cases]; NS – Not Significant; * denotes significance at < 0.05 level; 88 denotes significance at < 0.001 level.

## Discussion

Obesity is an established risk factor for most hormone-dependent cancers, including breast cancer. The pathology underlying this phenomenon may be related to the endocrine and metabolic profile of this state. In the present case–control study, our results indicate that metabolic changes among newly diagnosed breast cancer patients are consistent with a systemic stress response, possibly because of the presence and/or diagnosis of cancer activating the stress-system that, in turn, alters further the existing metabolic state to an environment conducive to tumor growth. It has been well established that stress, both acute and chronic induces a powerful cascade of immune, metabolic and inflammatory reactions [25]. On the other hand, we cannot exclude preexisting metabolic syndrome manifestations as a risk factor for the development of breast cancer and the two explanations are not mutually exclusive.

Our current data suggest that there is a positive association between triglycerides and ANG II levels in patients newly diagnosed with breast cancer. ANG II is a biologically active peptide of the renin-angiotensin system (RAS) involved in blood pressure regulation, tissue remodeling and angiogenesis, as well as in vascular and inflammatory pathologies. Consequently, the major functions attributed to ANG II (inflammation, angiogenesis and migration) are also related to cancer progression [26-28]. We also showed that there is a negative association of ANG II with HDL and aPAI which were in agreement with previous studies [29,30]. However, other studies reported a positive association between ANG II and breast cancer [31,32]. On the other hand, a very recent study involving more than 230,000 women (Swedish AMORIS Study) showed no association between HDL and breast cancer risk, while it demonstrated a weak protective association between circulating triglycerides and risk for breast cancer [33].

Obesity is increasingly associated with postmenopausal breast cancer risk [34], whereas, in premenopausal women there is an inverse relation between BMI and breast cancer risk [35,36]. We have previously demonstrated in our cross-sectional study that inflammatory biomarkers known to be elevated in breast cancer patients (IL-6 and CRP) are also increased in obese and insulin resistant pre-menopausal women [37]. The present findings, therefore, confirm that inflammatory markers, specifically CRP and TNF-α are elevated in newly diagnosed patients with breast cancer.

Inflammation is associated with poor prognosis and decreased survival in many cancers. As obesity *per se* is considered a subclinical inflammation, the increased levels of TNF-α and CRP in breast cancer cases in the present study are consistent with stress-induced inflammation among newly diagnosed breast cancer patients. In addition, CRP was positively correlated with BMI and inversely with adiponectin levels, in agreement with previous reports [22,23]. Thus, in obesity, the adipocytokines and in particular, adiponectin and the inflammatory mediators might exert an additive effect to positively impact breast cancer pathogenesis.

Our data showed significantly elevated mean level of serum glucose, diastolic blood pressure and reduced HDL in the breast cancer group. Previous studies reported that high fasting glucose levels were directly correlated with breast cancer both in pre-menopausal and postmenopausal women [38,39]. In addition, reduced HDL-cholesterol and increased blood pressure contributed to increased risk for breast cancer [40,41]. Furthermore, low HDL-cholesterol, hypertension, and hyperglycemia have all been associated with breast cancer [38,40,42-44].

The authors acknowledge some limitations. The case–control cross-sectional design limits the findings to at best, suggestive. The small sample size might explain the failure to produce significant associations in parameters that were expected to associate with clinical variables. Furthermore several confounders were excluded such as family history of breast cancer and medications and as such the findings cannot be generalized. Despite these limitations, the present study is among the few to observe pathologic changes in the adipocytokine, metabolic and immune biomarkers among early diagnosed breast cancer patients. These changes may reflect an earlier risk or a stressful environment conducive to tumor growth and/or both.

## Conclusions

In conclusion, inflammatory and metabolic changes are apparent among patients with early breast cancer as evidenced by the strong positive link between CRP and BMI, the positive association between ANG II and triglycerides, the negative association between HDL and adiponectin, and the strong negative association between PAI-1 and HDL. These associations, independent of age and BMI, are consistent with stress-induced changes secondary to the early breast cancer and/or the psychologic impact of the diagnosis, might enhance tumorigenic activity and lead to a poorer prognosis if left ignored.

## Competing interests

The authors declare no competing interests.

## Authors’ contributions

MSA and NMA conceived the study. AA, HMD, AAA and SY carried out data acquisition and interpretation. AMA and MSA analyzed the data and prepared the manuscript. SS and GPC drafted the revised and final version of the manuscript. All authors provided intellectual contributions to the manuscript and has read and approved the final version.

## Pre-publication history

The pre-publication history for this paper can be accessed here:

http://www.biomedcentral.com/1471-2407/13/54/prepub
